# Laparoscopic versus open distal pancreatectomy (LAPOP): study protocol for a single center, nonblinded, randomized controlled trial

**DOI:** 10.1186/s13063-019-3460-y

**Published:** 2019-06-13

**Authors:** Bergthor Björnsson, Per Sandström, Anna Lindhoff Larsson, Claes Hjalmarsson, Thomas Gasslander

**Affiliations:** 1 Department of Surgery and Clinical and Experimental Medicine, Linköping University, Linköping, Sweden; 2 Department of Surgery, Blekinge Hospital, Karlskrona, Sweden and Department of Clinical Sciences, Lund University, Lund, Sweden

**Keywords:** Minimally invasive, Laparoscopic, Distal pancreatectomy, Pancreatic surgery

## Abstract

**Background:**

Earlier nonrandomized studies have suggested that laparoscopic distal pancreatectomy (LDP) is advantageous compared with open distal pancreatectomy (ODP) regarding hospital stay, blood loss, and recovery. Only one randomized study has been conducted showing reduced time to functional recovery after LDP compared with ODP.

**Methods:**

LAPOP is a prospective randomized, nonblinded, parallel-group, single-center superiority trial. Sixty patients with lesions in the pancreatic body or tail that are found by a multidisciplinary tumor board to need surgical resection will be randomized to receive LDP or ODP. The primary outcome variable is postoperative hospital stay, and secondary outcomes include functional recovery (defined as no need for intravenous medications or fluids and as the ability of an ambulatory patient to perform activities of daily life), perioperative bleeding, complications, need for pain medication, and quality of life comparison.

**Discussion:**

The LAPOP trial will test the hypothesis that LDP reduces postoperative hospital stay compared with ODP.

**Trial registration:**

ISRCTN, 26912858. Registered on 28 September 2015.

**Electronic supplementary material:**

The online version of this article (10.1186/s13063-019-3460-y) contains supplementary material, which is available to authorized users.

## Background

A laparoscopic approach has been introduced for most abdominal procedures over the last 30 years, and a minimally invasive approach for these procedures has become the standard of care [1–5]. The main advantage expected from laparoscopic surgery is decreased patient discomfort and faster recovery, including a shortened postoperative hospital stay. A laparoscopic approach was described for resections of the pancreatic body and tail in the mid-1990s, but the general introduction of this method has been slow [6]. Before the publication of the only available randomized controlled trial comparing laparoscopic distal pancreatectomy (LDP) with open distal pancreatectomy (ODP), some cohort studies and systemic reviews suggested the benefits of the laparoscopic approach [7]. The benefits commonly mentioned are a shortening of hospital stay, reduction in blood loss and complications, and a higher rate of spleen-preserving procedures with LDP [8]. However, selection bias, such as differences in tumor size between the study groups, is commonly found in the available nonrandomized studies [8]. LDP has been in use for over 20 years without a single randomized controlled trial (RCT) supporting its proposed advantages. The LAPOP study was designed and initiated to investigate the hypothesis that a minimally invasive approach would shorten the hospital stay in a cohort of patients undergoing standard distal pancreatectomy [9]. A learning curve was passed and described prior to starting the study [10]. Since the start of the LAPOP study, one RCT using a similar approach has been published, and the results suggest enhanced functional recovery and a shortening of hospital stay using the laparoscopic approach [7].

## Methods

### Design and patients

The LAPOP study is a randomized, controlled, nonblinded, parallel assignment, single-center superiority trial of the effectiveness of LDP versus ODP in the settings of lesions in the pancreatic body or tail, regardless of suspicion of malignancy. Patients who fulfill the inclusion criteria and do not satisfy any of the exclusion criteria will be randomized in a 1:1 manner to LDP or ODP.

All patients with tumors in the body or tail of the pancreas in the South-East health district in Sweden are presented to the multidisciplinary board (MDB) at the study site (Linköping University Hospital), where eligibility screening is performed.

### Sample size

The sample size considerations are based on the primary endpoint hospital stay in an intention-to-treat manner. A one-sided power calculation is used because none of the previous publications indicated an inferiority for LDP. The assumed mean hospital stay is 5 and 7.5 days for LDP and ODP, respectively. The standard deviation is 3.5 with a type I error = 0.05, and 25 patients are needed in each group to achieve a 0.8 power. Thirty patients will be included in each group to include possible drop outs.

### Inclusion and exclusion criteria

The following inclusion criteria are used for the LAPOP study: 1) patients with lesion in the body or tail of the pancreas who require surgery (indication set by multidisciplinary conference); 2) operable patient (based on the local preoperative evaluation); 3) possibility to achieve an R0 resection without resection of additional organs (besides the spleen); 4) patients with a performance status of 0–2 according to the World Health Organization (WHO) scale; 5) written informed consent provided; and 6) age > 18 years.

The following exclusion criteria will be used: 1) pregnancy and/or lactation; 2) patients unable to comply with the protocol because of language or cognitive function; 3) preoperatively defined need to resect organs other than the pancreas and spleen; 4) preoperatively defined division line of the pancreas to the right of the superior mesenteric vein.

### Randomization and inclusion

Randomization will be performed using computer-generated random numbers in blocks of 10 (5:5). A research nurse who will not participate in patient care will perform the randomization. Study group allocation will depend on the contents of sealed opaque envelopes generated in the above-described manner, and envelopes will be opened after patient inclusion. A surgeon in the outpatient clinic will provide written and oral information about the study, and patients who agree to participate will sign a written informed consent.

### Treatment

#### LDP (treatment group)

Patients will receive general anesthesia in a supine position, and four trocars will be placed: one above the umbilicus (12 mm); one in the lateral part of the left rectus abdominis muscle (12 mm); one to the left of the xiphoid process (5 mm); and one in the left flank (5 mm). The surgeon and assisting surgeon (controlling the camera) will stand on the patient’s right side.

The left colonic flexure will be mobilized, and the splenocolic ligament will be divided. Thereafter, the omental bursa will be opened, and the stomach completely mobilized, including the short gastric vessels. The lesion in the pancreas will be identified with or without the help of ultrasonography. The inferior border of the pancreas will be dissected, and a band placed around the pancreas between the lesion and spleen if appropriate. A band will be placed around the pancreas to the right of the lesion (and the splenic vein if splenectomy is intended). Before dividing the pancreas, the splenic artery will be identified and secured using Hem-o-lock clips (Teleflex Medical, Weck Drive, Research Triangle Park, NC, USA). In cases of spleen-preserving procedures, the splenic artery will be dissected from the pancreas and left intact. To improve visibility of the superior border of the pancreas, the stomach will be sutured to the anterior abdominal wall. Depending on the preoperative assessment, lymphadenectomy will be performed as indicated for pancreatic adenocarcinoma. The pancreas will be divided using a linear stapler with a cartridge size based on the thickness of the pancreas. A gradual stepwise compression technique and division will be used as described previously to reduce the risk of rupture of the pancreas along the stapling line [11]. Following division of the pancreas, the resection will be performed in a medial to lateral direction. The surgical specimen will be placed in a plastic bag and retrieved through enlargement of the trocar incision above the umbilicus. A 24 CH passive drain will be placed through the trocar incision in the left flank with the tip in front of the pancreatic transection line.

#### ODP (control group)

A midline laparotomy will be performed, retractors placed, and the resection performed essentially as described above. The stomach will be retracted with the retractor instead of sutures, and the splenic artery will be suture ligated instead of using clips. No attempt will be made to dissect around the pancreas between the lesion and the spleen as antegrade resection is applied. The division of the pancreas will be performed in the same manner as the LDP group, and a drain placed in the same manner. Lymphadenectomy will be performed when indicated.

### Conversion to open surgery

Conversion (in the LDP group) is defined as any incision that is not for trocar placement or surgical specimen retrieval. In cases of conversion, analyses will be performed in an intention-to-treat manner.

### Postoperative treatment

Both groups follow a fast-track program that omits the use of a nasogastric tube directly after surgery (in the operating theatre) and encourages oral intake as soon as possible. Epidural anesthesia is allowed in the ODP group but not in the LDP group.

### Study outcomes

The primary outcome variable of the LAPOP study is hospital stay, which is defined as the number of days spent in the hospital after surgery. The study hypothesis is that LDP results in shorter hospital stays than ODP.

Secondary outcomes include functional recovery (key secondary outcome), which is defined as the number of days needed to reach no need for intravenous drug administration or fluids and ambulatory patients who are able to perform activities of daily living (ADL). These outcomes do not exclude discharge with drains or urinary catheters. Other secondary outcomes are perioperative bleeding (assessed by an anesthesia nurse), use of pain medications, complications (according to Clavien Dindo classification), frequency of postoperative pancreatic fistula, quality of life, lymph node harvesting, R0 frequency, and cost.

### Follow-up and data collection

**Fig. 1:**
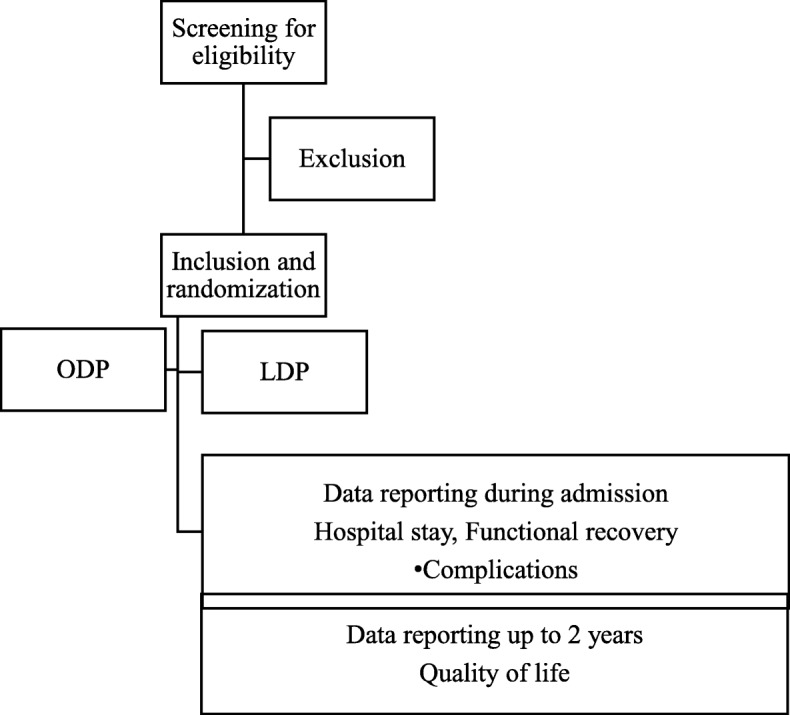
Trial flow diagram. LDP laparoscopic distal pancreatectomy, OPD open distal pancreatectomy

Baseline characteristics will be collected using standardized case report forms (CRFs) before randomization. Data for the outcome variables will be collected on separate CRFs for surgical procedure, hospital stay, pathology report, and 90-day follow up. Quality of life will be assessed using the EORTC QLQ-C30 with the addition of the PAN26 module and EQ-5D (Fig. 1).

Study subjects will complete the questionnaires at inclusion, 4–6 weeks after surgery, and 6, 12, and 24 months postoperatively. A research nurse will contact patients who do not return questionnaires to improve follow-up. Survival will be followed up to 24 months postoperatively (Table 1).

**Table 1:** Schedule of enrollment, interventions, and assessments

	MTB	Outpatient clinic	Surgery	POD1	POD2	POD3	Discharge	4–6 weeks postop	POD90	6 months postop	12 months postop	24 months postop
Screening for eligibility	x											
Informed consent		x										
Randomization		x										
Baseline characteristics		x										
Intraoperative outcomes			x									
Postoperative outcomes				x	x	x	x		x			
QOL assessment		x						x		x	x	x

*MTB* multidisciplinary tumor board, *POD* postoperative day, *postop* postoperatively, *QOL* quality of life

### Statistical analysis

Analyses will be based on the intention-to-treat principle. Primary outcome and key secondary outcomes will be tested using *t* tests. Demography, treatment, and clinical data will be reported.

Quality of life will be measured using the EORTC QLQ-C30, EORTC PAN26, and EQ-5D for analyses.

Cost-benefit analysis will be performed for the surgery, postoperative complications, and interventions, days in hospital, need for postoperative outpatient treatment, readmissions for complications, and tumor recurrence. The costs for sick leave will be included.

The study is performed without a data monitoring committee (DMC), and no interim analysis is planned. The adherance to the CONSORT checklist is shown as Additional file 1.

## Discussion

Despite its increasing use, LDP has only been compared with ODP in a single randomized trial of 108 patients after a structured nationwide implementation of the method [7, 12]. The results from the LEOPARD trial support the hypothesis that LDP shortens hospital stay, but this finding must be further investigated in different settings to increase the generalizability of the high-level evidence available. Functional recovery may be a more reliable endpoint than hospital stay in multicenter studies, and particularly multinational studies, but it remains partially subjective. Our study is performed in a homogenous population that represents approximately 10% of the Swedish nation. Therefore, differences in routines should not influence the results, and other factors that can influence the length of stay should be equally distributed between the groups due to the randomized nature of the study. Therefore, the more robust endpoint of hospital stay is more suitable as the primary endpoint rather than functional recovery. Due to the structure of the Swedish healthcare system, all patients from the South-east health district (approximately 1 million inhabitants) will be eligible for the study and evaluated at the same tumor board. The study includes all types of tumors in adult patients with no upper limit of tumor size as long as standard distal pancreatectomy may be used. Therefore, the results will be useful for general decision making. The secondary outcomes will provide information on health economic aspects and quality of life.

This study has several limitations that should be acknowledged. Blinding is impractical and will not be used because Swedish patients have access to their health records, including operation notes, and the unit performing the study is small in size. The lack of blinding may be a limitation, but the importance of blinding in surgical studies is largely unknown. The use of fixed size blocks for randomization makes it theoretically possible to know beforehand the allocation of every tenth patient randomized. However, the practical implications of this limitation are minor because of the organization, where the decision to offer participation is made at a multidisciplinary conference based on rigid inclusion and exclusion criteria, and a research nurse informs the patients at the outpatient clinic prior to randomization.

## Conclusion

The LAPOP trial will provide level 1 evidence on hospital stay after LDP vs. ODP and increase the level of evidence for guidelines on the use of LDP in the future.

## Trial status

The current protocol version is 1.1 as of 13 September 2015. The randomization began (recruitment) on 11 May 2015, and 53 (88%) of 60 patients were randomized at the time of manuscript submission (31 October 2018). Therefore, the trial is ahead of schedule, and recruitment is expected to end in May 2019.

## Additional file

Additional file 1: CONSORT checklist. (DOCX 30 kb)

## Data Availability

Datasets are not available due to the ongoing recruitment.

## Abbreviations

CRF: Case report form
LDP: Laparoscopic distal pancreatectomy
ODP: Open distal pancreatectomy
WHO: World Health Organization

## References

[1] Gagner M, Lacroix A, Bolte E (1992). Laparoscopic adrenalectomy in Cushing's syndrome and pheochromocytoma. N Engl J Med.

[2] Hashizume M, Sugimachi K, Ueno K (1992). Laparoscopic splenectomy with an ultrasonic dissector. N Engl J Med.

[3] Gagner M, Pomp A, Heniford BT, Pharand D, Lacroix A (1997). Laparoscopic adrenalectomy: lessons learned from 100 consecutive procedures. Ann Surg.

[4] Jacobs JK, Goldstein RE, Geer RJ (1997). Laparoscopic adrenalectomy. A new standard of care. Ann Surg.

[5] Katkhouda N, Hurwitz MB, Rivera RT, Chandra M, Waldrep DJ, Gugenheim J (1998). Laparoscopic splenectomy: outcome and efficacy in 103 consecutive patients. Ann Surg.

[6] Gagner M, Pomp A, Herrera MF (1996). Early experience with laparoscopic resections of islet cell tumors. Surgery..

[7] de Rooij T, van Hilst J, van Santvoort H, Boerma D, van den Boezem P, Daams F (2019). Minimally Invasive Versus Open Distal Pancreatectomy (LEOPARD): a multicenter patient-blinded randomized controlled trial. Ann Surg..

[8] Mehrabi A, Hafezi M, Arvin J, Esmaeilzadeh M, Garoussi C, Emami G (2015). A systematic review and meta-analysis of laparoscopic versus open distal pancreatectomy for benign and malignant lesions of the pancreas: it's time to randomize. Surgery..

[9] Hartwig W, Vollmer CM, Fingerhut A, Yeo CJ, Neoptolemos JP, Adham M (2014). Extended pancreatectomy in pancreatic ductal adenocarcinoma: definition and consensus of the International Study Group for Pancreatic Surgery (ISGPS). Surgery..

[10] Hasselgren K, Halldestam I, Fraser MP, Benjaminsson Nyberg P, Gasslander T, Bjornsson B (2016). Does the introduction of laparoscopic distal pancreatectomy jeopardize patient safety and well-being?. Scand J Surg.

[11] Asbun HJ, Stauffer JA (2011). Laparoscopic approach to distal and subtotal pancreatectomy: a clockwise technique. Surg Endosc.

[12] de Rooij T, van Hilst J, Boerma D, Bonsing BA, Daams F, van Dam RM (2016). Impact of a Nationwide Training Program in Minimally Invasive Distal Pancreatectomy (LAELAPS). Ann Surg.

