# Supplementary material for: Laparoscopic versus open distal pancreatectomy (LAPOP): study protocol for a single center, nonblinded, randomized controlled trial
Source: Trials. 2019 Jun 13;20:356. doi: 10.1186/s13063-019-3460-y (PMC6567450; doi:10.1186/s13063-019-3460-y)
Supplement: Supplementary file 1 — CONSORT checklist. (DOCX 30 kb) [file 13063_2019_3460_MOESM1_ESM.docx]

**Item 1: Descriptive title identifying the study design, population, interventions, and, if applicable, trial acronym**

Laparoscopic versus open distal pancreatectomy (LAPOP), study protocol for a single centre, non-blinded randomized controlled trial

Found on page 1 of the manuscript

**Item 2a: Trial identifier and registry name. If not yet registered, name of intended registry**

Registration: ISRCTN:26912858, date of registration 28/9 2015

Found on page 2 of the manuscript

**Item 2b: All items from the World Health Organization Trial Registration Data Set**

| **Data category** | **Information**[**^32^**](http://www.spirit-statement.org/spirit-statement/references#32) |
| --- | --- |
| Primary registry and trial identifying number | www.isrctn.com ISRCTN26912858  Found on page 2 of the manuscript |
| Date of registration in primary registry | 28 September, 2015  Found on page 2 of the manuscript |
| Secondary identifying numbers | None |
| Source(s) of monetary or material support | The County Counicil of Östergötland, a grant was received from the Medical Research Council of Southeast Sweden  Found on page 9 of the manuscript |
| Primary sponsor | The County Counicil of Östergötland  Found on page 9 of the manuscript |
| Secondary sponsor(s) | None |
| Contact for public queries | *Bergthor Björnsson*, MD, PhD, +46703766890, [bergthor.bjornsson@liu.se](mailto:bergthor.bjornsson@liu.se)  Found on page 1 of the manuscript |
| Contact for scientific queries | *Bergthor Björnsson*, MD, PhD, +46703766890, [bergthor.bjornsson@liu.se](mailto:bergthor.bjornsson@liu.se)  Found on page 1 of the manuscript |
| Public title | Comparison of laparoscopic and open distal pancreatectomy  Not provided in the manuscript |
| Scientific title | Laparoscopic versus open distal pancreatectomy (LAPOP), study protocol for a single centre, non-blinded randomized controlled trial  Found on page 1 of the manuscript |
| Countries of recruitment | Sweden  ound on page 1 of the manuscript |
| Health condition(s) or problem(s) studied | Tumors in the pancreatic body and tail  Found on page 3 of the manuscript |
| Intervention(s) | Study group: laparoscopic distal pancreatectomy, control group: open distal pancreatectomy  Found on pages 5-6 of the manuscript |
| Key inclusion and exclusion criteria | Ages eligible for study: ≥18 years Sexes eligible for study: both Accepts healthy volunteers: no Inclusion criteria: adult patient (≥ 18 years), with tumor in the body or tail of the pancreas requiring surgical resection Exclusion criteria: pregnancy/lactation, need of extended resection of other organs than the pancreas/spleen, unfit for surgery, unable to give informed consent  Found on page 4 of the manuscript |
| Study type | Interventional Allocation: randomized Intervention model: parallel assignment Masking: none  Found on page 3 of the manuscript |
| Date of first enrolment | 5/11 2015  Found on page 9 of the manuscript |
| Target sample size | 60  Found on pages 4 and 9 of the manuscript |
| Recruitment status | Recruiting  Found on page 9 of the manuscript |
| Primary outcome(s) | Hospital stay  Found on page 6 of the manuscript |
| Key secondary outcomes | Functional recovery  Found on page 6 of the manuscript |

**Item 3: Date and version identifier**

2015-09-13, version 1.1

Found on page 9 of the manuscript

**Item 4: Sources and types of financial, material, and other support.**

The LAPOP study is investigator initiated, a grant was received from the Medical Research Council of Southeast Sweden

Found on page 9 of the manuscript

**Item 5a: Names, affiliations, and roles of protocol contributors**

BB initiated the study, drafted the protocol and the manuscript. BB,PS,ALL,CH and TG contributed to the final design of the study. BB performed sample size calculations. BB,PS,ALL,CH and TG will contribute to data sampling and analysis. BB wrote the manuscript, PS, ALL, CH and TG have critically reviewed the manuscript and are fully aware of the publication.

Found on page 9 of the manuscript

**Item 5b: Name and contact information for the trial sponsor**

Region Östergötland, c/o dr. Bärbel Jung, Linköping University Hospital Surgical clinic. Garnesonvägen 58185 Linköping, Sweden.

Found on page 9 of the manuscript

**Item 5c: Role of study sponsor and funders, if any, in study design; collection, management, analysis, and interpretation of data; writing of the report; and the decision to submit the report for publication, including whether they will have ultimate authority over any of these activities.**

The funding source had no role in the design of this study and will not have any role during its execution, analyses, interpretation of the data, or decision to submit results.

Found on page 9 of the manuscript

**Item 5d: Composition, roles, and responsibilities of the coordinating centre, steering committee, endpoint adjudication committee, data management team, and other individuals or groups overseeing the trial, if applicable (see** [**Item 21a**](http://www.spirit-statement.org/formal-committee/) **for Data Monitoring Committee)**

Due to the small size of the LAPOP study and the single center design no committees have been appointed but all listed authors are included in the necessary discussions and decisions.

Not included in the manuscript

**Item 6a: Description of research question and justification for undertaking the trial, including summary of relevant studies (published and unpublished) examining benefits and harms for each intervention.**

Included in the “Background” chapter of the manuscript

Found on page 3 of the manuscript

**Item 6b: Explanation for choice of comparators**

Described in the manuscript, the only available comparator is used in this study

Found on page 3 of the manuscript

**Item 7: Specific objectives or hypotheses**

Included in the manuscript

Found on page 6 of the manuscript

**Item 8: Description of trial design including type of trial (e.g., parallel group, crossover, factorial, single group), allocation ratio, and framework (e.g., superiority, equivalence, non-inferiority, exploratory)**

“The LAPOP study is a randomized controlled non-blinded parallel assignment single-center superiority trial…”

Found on page 3 of the manuscript

**Item 9: Description of study settings (e.g., community clinic, academic hospital) and list of countries where data will be collected. Reference to where list of study sites can be obtained.**

The study is conducted at Linköping University Hospital, Sweden.

Found on page 3 of the manuscript

**Item 10: Inclusion and exclusion criteria for participants. If applicable, eligibility criteria for study centres and individuals who will perform the interventions (e.g., surgeons, psychotherapists)**

The inclusion criteria for the LAPOP study are set as follows:

Patients with lesion in the body or tail of the pancreas demanding surgery (indication set by multidisciplinary conference).

Operable patient (as the local preoperatively evaluation dictates).

Possibility to achieve R0-resection without resection of additional organs (besides the spleen)

Patients with performance status 0-2 according to WHO scale.

Written informed consent.

Age > 18 years

The following exclusion criteria apply:

Pregnancy and/or lactation.

Patients being unable to comply with the protocol for reasons of language or cognitive function.

Preoperatively defined need to resect other organs than pancreas and spleen.

Preoperatively defined division line of pancreas to the right of the superior mesenteric vein

Found on page 4 of the manuscript

**Item 11a: Interventions for each group with sufficient detail to allow replication, including how and when they will be administered**

LDP (treatment group)

In general anesthesia with the patient in the supine position 4 trocars are placed; above the umbilicus (12 mm), in the lateral part of the left rectus abdominis muscle (12 mm), to the left of the xiphoid process (5 mm) and in the left flank (5 mm). The surgeon and assisting surgeon (controlling the camera) stand on the patients right side.

The left colonic flexure is mobilized and the splenocolic ligament divided, after that the omental bursa is opened and the stomach completely mobilized, including the short gastric vessels. The lesion in the pancreas is identified with or without the help of ultrasonography. The inferior border of the pancreas is dissected and if found appropriate a band is placed around the pancreas between the lesion and the spleen. To the right of the lesion a band is placed around the pancreas (and the splenic vein if splenectomy is intended). Before dividing the pancreas the splenic artery is identified and secured with Hem-o-lock clips (Teleflex Medical, Weck Drive, Research Triangle Park, NC, USA). In case of spleen preserving procedure the splenic artery is dissected from the pancreas and left intact. In order to improve visibility of the superior border of the pancreas the stomach is sutured to the anterior abdominal wall. Depending on the preoperative assessment lymphadenectomy is performed as indicated for pancreatic adenocarcinoma. The pancreas is divided using linear stapler with cartridge size based the the thickness of the pancreas. A slow compression and division is applied in order to reduce risk of rupture of the pancreas along the stapling line. Following division of the pancreas the resection as carried out in a medial to lateral direction. The surgical specimen is placed in a plastic bag and retrieved through enlargement of the trocar incision above the umbilicus. A 24 Ch passive drain is placed through the trocar incision in the left flank with the tip in front of the pancreatic transection line.

ODP (control group)

After a midline laparotomy and placement of retractors the resection is carried out essentially as described above. The stomach is retracted with retractor instead of sutures and the splenic artery is suture ligated instead of the use of clips. No attempt is made to dissect around the pancreas between the lesion and the spleen as antegrade resection is applied. The division of the pancreas is performed in the same manner as in the LDP group and drain is placed in the same way.

Found on pages 5-6 of the manuscript

**Item 11b: Criteria for discontinuing or modifying allocated interventions for a given trial participant (e.g., drug dose change in response to harms, participant request, or improving/worsening disease)**

Patients allocated to the laparoscopic group may undergo conversion to open surgery if the surgeon finds it appropriate. This is not subject to criteria and the case will be handled as intention to treat in the results of the study

Found on page 6 of the manuscript

**Item 11c: Strategies to improve adherence to intervention protocols, and any procedures for monitoring adherence (e.g., drug tablet return; laboratory tests)**

As the patients are allocated to treatment that is given under general anesthesia this is not applicable. For the follow up, in particular the quality of life questioners research nurse will contact the participants telephonically if the questioners are not returned.

Found on page 7 of the manuscript

**Item 11d: Relevant concomitant care and interventions that are permitted or prohibited during the trial.**

As the study intervention is one surgical intervention this item is not applicable.

Not included in the manuscript

**Item 12: Primary, secondary, and other outcomes, including the specific measurement variable (e.g., systolic blood pressure), analysis metric (e.g., change from baseline, final value, time to event), method of aggregation (e.g., median, proportion), and time point for each outcome. Explanation of the clinical relevance of chosen efficacy and harm outcomes is strongly recommended.**

Study outcomes

The primary outcome variable of the LAPOP study is hospital stay defined as the number of days spent in the hospital after surgery. The study hypothesis is that LDP results in shorter hospital stay than ODP.

Secondary outcomes include functional recovery (key secondary outcome) defined as the number of days needed to reach no need for intravenous drug administration or fluids as well ambulatory patient able to perform ADL. This does not exclude discharge with drains or urinary catheters. Other secondary outcomes are perioperative bleeding, use of pain medications, complications (according to Clavien Dindo classification), the frequency of postoperative pancreatic fistula, quality of life and, lymph node harvesting, R0 frequency and cost.

Found on page 7 of the manuscript

**Item 13: Time schedule of enrolment, interventions (including any run-ins and washouts), assessments, and visits for participants. A schematic diagram is highly recommended**

|  | MTB | Outpatient clinic | Surgery | POD1 | POD2 | POD3… | Discharge | 4-6 weeks postop | POD90 | 6 months postop | 12 months postop | 24 months postop |
| --- | --- | --- | --- | --- | --- | --- | --- | --- | --- | --- | --- | --- |
| Screening for eligibility | x |  |  |  |  |  |  |  |  |  |  |  |
| Informed consent |  | x |  |  |  |  |  |  |  |  |  |  |
| Randomisation |  | x |  |  |  |  |  |  |  |  |  |  |
| Baseline characteristics |  | x |  |  |  |  |  |  |  |  |  |  |
| Intraop outcomes |  |  | x |  |  |  |  |  |  |  |  |  |
| Post op outcomes |  |  |  | x | x | x | x |  | x |  |  |  |
| QOL assessment |  | x |  |  |  |  |  | x |  | x | x | x |

Found as an additional file

**Item 14: Estimated number of participants needed to achieve study objectives and how it was determined, including clinical and statistical assumptions supporting any sample size calculations.**

The sample size considerations are based on the primary endpoint hospital stay in an intention to treat manner. One sided power calculation is used as none of the previous publications indicates inferiority for LDP. Assumed mean hospital stay is 5 and 7.5 days for laparoscopic respective open operations. The standard deviation is 3,5 and with type I error = 0.05 and a 0.8 power 25 patients are needed in each group.

The sample size is calculated based on a expected conversion rate of 20%

Due to possible drop-outs 30 patients will be inculded in each group.

Found on page 4 of the manuscript

**Item 15: Strategies for achieving adequate participant enrolment to reach target sample size.**

All patients with tumors in the body and the tail of the pancreas in the South-East health district in Sweden are presented on the MDB (Multidisciplinary board) at the study site. Here eligibility screening is performed.

Found on page 3 of the manuscript

**Item 16a: Method of generating the allocation sequence (e.g., computer-generated random numbers), and list of any factors for stratification. To reduce predictability of a random sequence, details of any planned restriction (e.g., blocking) should be provided in a separate document that is unavailable to those who enrol participants or assign interventions.**

Randomisation will be performed with computer-generated random numbers in block of 10 (5:5). The randomisation is done by a research nurse that does not participate in the patient care.

Found on pages 4-5 of the manuscript

**Item 16b: Mechanism of implementing the allocation sequence (e.g., central telephone; sequentially numbered, opaque, sealed envelopes), describing any steps to conceal the sequence until interventions are assigned.**

Study group allocation will depend upon the contents of sealed opaque envelopes generated in this manner, opened after the patient inclusion.

Found on page 5 of the manuscript

**Item 16c: Who will generate the allocation sequence, who will enrol participants, and who will assign participants to interventions.**

The allocation is computer-generated by a research nurse, the patients are enrolled by surgeons. The next envelope (in numeric order) is opened when the patient has accepted to participate in the study.

Found on pages 4-5 of the manuscript

**Item 17a: Who will be blinded after assignment to interventions (e.g., trial participants, care providers, outcome assessors, data analysts), and how.**

The study is un-blinded

Found on page 3 of the manuscript

**Item 17b: If blinded, circumstances under which unblinding is permissible, and procedure for revealing a participant’s allocated intervention during the trial.**

Not applicable as the study is not blinded

Not included in the manuscript

**Item 18a: Plans for assessment and collection of outcome, baseline, and other trial data, including any related processes to promote data quality (e.g., duplicate measurements, training of assessors) and a description of study instruments (e.g., questionnaires, laboratory tests) along with their reliability and validity, if known. Reference to where data collection forms can be found, if not in the protocol.**

Outcomes based on laboratory values (pancreatic fistula) will be measured in accredited clinical laboratory at Linköping university hospital, the questioners used are validated and have been used in multiple previous studies.

Not included in the manuscript

**Item 18b: Plans to promote participant retention and complete follow-up, including list of any outcome data to be collected for participants who discontinue or deviate from intervention protocols.**

For the follow up, in particular the quality of life questioners research nurse will contact the participants telephonically if the questioners are not returned.

Found on page 7 of the manuscript

**Item 19: Plans for data entry, coding, security, and storage, including any related processes to promote data quality (e.g., double data entry; range checks for data values). Reference to where details of data management procedures can be found, if not in the protocol.**

Data is collected on paper case report forms that are kept by the principal investigator separate from a list of participants. At the end of 90 day follow up for the last included patient the data will be entered into an electronic database secured by password and without reference to the patients included. After last follow up (2 years) the list of participants will be destroyed.

Not included in the manuscript

**Item 20a: Statistical methods for analysing primary and secondary outcomes. Reference to where other details of the statistical analysis plan can be found, if not in the protocol.**

The analyses will be based on the intention to treat principle. Primary outcome and key secondary outcome will be tested with t-test. Demography, treatment and clinical data will be reported.

Quality of life as measured by the EORTC QLQ-C30, EORTC PAN26 and EQ-5D will be analysed.

Cost-benefit analysis will be performed with regards to the operation, postoperative complications and interventions, days in hospital, need for postoperative outpatient treatment, readmissions for complications, tumor recurrence. Cost for sick leave will be included.

Found on page 7 of the manuscript

**Item 20b: Methods for any additional analyses (e.g., subgroup and adjusted analyses).**

Nu further analysis are planned.

Not included in the manuscript

**Item 20c: Definition of analysis population relating to protocol non-adherence (e.g., as randomised analysis), and any statistical methods to handle missing data (e.g., multiple imputation).**

Analysis will be done as intention to treat, missing data will be omitted from analysis

Found on page 7 of the manuscript

**Item 21a: Composition of data monitoring committee (DMC); summary of its role and reporting structure; statement of whether it is independent from the sponsor and competing interests; and reference to where further details about its charter can be found, if not in the protocol. Alternatively, an explanation of why a DMC is not needed.**

The study is done without DMC as the number of planned participants does not allow for meaningful interim analysis.

Found on page 7 of the manuscript

**Item 21b: Description of any interim analyses and stopping guidelines, including who will have access to these interim results and make the final decision to terminate the trial.**

No interim analysis is planned

Found on page 7 of the manuscript

**Item 22: Plans for collecting, assessing, reporting, and managing solicited and spontaneously reported adverse events and other unintended effects of trial interventions or trial conduct.**

The study outcomes (secondary) includes complications as graded by the Clavien Dindo scale, these will be reported

Found on page 7 of the manuscript

**Item 23: Frequency and procedures for auditing trial conduct, if any, and whether the process will be independent from investigators and the sponsor.**

The trial will not be audited

Not included in the manuscript

**Item 24: Plans for seeking research ethics committee/institutional review board (REC/IRB) approval**

The study protocol was approved by the regional ethics board in Linköping on June 10^th^ 2015. The number assigned to the study is 2015/39-31

Found on page 9 of the manuscript

**Item 25: Plans for communicating important protocol modifications (e.g., changes to eligibility criteria, outcomes, analyses) to relevant parties (e.g., investigators, REC/IRBs, trial participants, trial registries, journals, regulators)**

Any major change to the protocol will demand revision by the ethics board as a formal application and therefore such changes will be sent to the board.

Not included in the manuscript

**Item 26a: Who will obtain informed consent or assent from potential trial participants or authorised surrogates, and how**

Written and oral information about the study will be given by surgeon at the outpatient clinic and if the patient accepts to participate this is followed by a written informed consent

Found on page 5 of the manuscript

**Item 26b: Additional consent provisions for collection and use of participant data and biological specimens in ancillary studies, if applicable.**

The study questions are described above as primary and secondary outcomes, no other analysis are planned

Not included in the manuscript

**Item 27: How personal information about potential and enrolled participants will be collected, shared, and maintained in order to protect confidentiality before, during, and after the trial.**

In accordance with Swedish regulation for ethical approval for this study the information gathered will be stored by the responsible principal investigator and not disseminated further. All material is decoded with regards to individual patients and the key for this stored separately.

Not included in the manuscript

**Item 28: Financial and other competing interests for principal investigators for the overall trial and each study site.**

None

Found on page 9 of the manuscript

**Item 29: Statement of who will have access to the final trial dataset, and disclosure of contractual agreements that limit such access for investigators.**

The principal investigator as those investigators listed in the ethical application will have access to the final dataset

Not included in the manuscript

**Item 30: Provisions, if any, for ancillary and post-trial care, and for compensation to those who suffer harm from trial participation.**

None

Not included in the manuscript

**Item 31a: Plans for investigators and sponsor to communicate trial results to participants, healthcare professionals, the public, and other relevant groups (e.g., via publication, reporting in results databases, or other data sharing arrangements), including any publication restrictions.**

The results of the study will be submitted to a peer reviewed medical journal.

Not included in the manuscript

**Item 31b: Authorship eligibility guidelines and any intended use of professional writers.**

Investigators that fulfill the criteria for authorship will be included as authors, no professional writers will participate in the process

Not included in the manuscript

**Item 31c: Plans, if any, for granting public access to the full protocol, participant-level dataset, and statistical code.**

Not planned

Found on page 9 of the manuscript

**Item 32: Model consent form and other related documentation given to participants and authorised surrogates.**

Not included as the document is in Swedish and has been reviewed and accepted by the ethical committee

Not included in the manuscript

**Item 33: Plans for collection, laboratory evaluation, and storage of biological specimens for genetic or molecular analysis in the current trial and for future use in ancillary studies, if applicable.**

Not applicable

Not included in the manuscript
